# Risk assessment and spatio-temporal distribution of dissolved trace metals in Swarna, Sharavati and Kali estuaries, South-West Coast of India

**DOI:** 10.1007/s11356-022-22812-4

**Published:** 2022-09-06

**Authors:** D’Souza Nishitha, Athiyarath Krishnan Sudheer, Kumar Arun, Vadakkeveedu Narayan Amrish, Gaddam Mahesh, Harikripa Narayana Udayashankar, Keshava Balakrishna

**Affiliations:** 1 Department of Civil Engineering, Manipal Institute of Technology, Manipal Academy of Higher Education, Manipal, 576 104 India; 2 Geosciences Division, Physical Research Laboratory, Navrangpura, Ahmedabad 380009 India

**Keywords:** Dissolved trace metals, Salinity gradient, Risk assessment, Environmental pollutants, Ecosystem, Hazard Index

## Abstract

Trace metals act as a limiting nutrient and prerequisite for primary productivity in marine environments. The distribution of metals in dissolved phase along the salinity gradients of Swarna, Sharavati and Kali estuaries in southwestern India, during post and pre-monsoon seasons, were studied. We have investigated the behaviour of trace metals in the estuarine environment and their extent of impact on human health and ecosystem. The study revealed, non-conservative behaviour of dissolved Mn, Fe, Ni, Cd and Co in the estuaries. Whereas Cu behaved non-conservatively in post-monsoon and conservatively in pre-monsoon seasons. Risk assessment studies revealed that higher chronic daily intake (CDI) in humans, through dermal pathway, in Swarna and Sharavati estuaries during post-monsoon, whereas it was during pre-monsoon season in the Kali estuary. Hazard Index values for the studied metals in adults and children are below risk thresholds, though children are more prone to health risk through the dermal pathway.

**Supplementary Information:**

The online version contains supplementary material available at 10.1007/s11356-022-22812-4.

## Introduction

Aquatic systems like rivers and lakes have a long history of human habitation and resulting anthropogenic activities surrounding them. This has caused a significant impact on these systems, causing environmental problems (Bryan et al. 1980; Langston 1982; Salas et al. 2017; Wang et al. 2018; Jia et al. 2021). The continentally mobilised material finally reaches the world oceans through different transport pathways. These include external (atmospheric deposition, rivers, industrial and municipal effluents) and internal (remobilisation from the benthic and resuspension of the sediments) sources (Shim et al. 2012). Before reaching the open ocean, the material will pass through the estuarine zone, where it gets significantly modified. Thus, estuaries with extreme hydrodynamic conditions serve as an interface between fresh water and marine ecosystems and play a crucial role in transporting the land-sourced pollutants to the open sea (Liu et al. 2019; Celis-Hernandez et al. 2021; Lu et al. 2021).

The changes in the estuarine environment are temporal which occur due to hourly (tide) to seasonal variations (river discharge). Any modifications in the hydrological regime such as a river, tide, waves, currents and sediment discharge affect the estuaries, which changes the previously established equilibrium (Avinash et al. 2012). The strong gradients in this transition zone cause constant variations in the chemical variables such as pH, temperature, dissolved oxygen and the amount of particulates which result from the mixing of fresh water and seawater (Sun et al. 2018; Karbassi and Heidari 2015). Furthermore, in the areas of high turbidity zones, these changing gradients stimulate biogeochemical processes, resulting in increased desorption and sorption of pollutants. Thus, various contaminants undergo dynamic changes to their environmental fate and behaviour in the marine environment.

Among the various inorganic contaminants entering the marine environments via estuaries, trace metals serve as one of the priority pollutants because of their persistent nature, toxicity and ability to accumulate in the organisms (Bhattacharya et al. 2015; Mitra et al. 2018; Truchet et al. 2021; Niu et al. 2021; Jia et al. 2021). Processes such as resuspension and turbidity currents lead to diffusing dissolved components from the sediments and mixing with the seawater (Cantwell et al. 2008). Moreover, the distribution and behaviour of the dissolved trace metals are a function of complex dynamics in the estuary (Sanchez et al., 2004). For example, the metal Fe is always found complexed with the dissolved organic matter in the form of colloidal phase in the river water (Wen et al. 1999). Whereas, during estuarine mixing, the seawater cations neutralise the colloidal dissolved organic matter resulting in colloidal Fe flocculation, which increases Fe removal from the water column to sediments and decreases Fe input to the oceans (Boyle et al. 1977; Sholkovitz et al. 1978; Hunter et al. 1997; Stolpe and Hassellöv, 2007; Fang and Wang 2022). In contrast, metal Cd gets desorbed from the suspended particles during the mixing as a result of the formation of the chloro-complexes in water. Thus, trace metals can be added, removed or modified along estuarine gradients due to a variety of complex biological, physical and chemical transformation and remineralisation processes. However, increased metal concentration in the water column is more toxic than in the sediments as it is easily ingested by the organisms, which can cause a direct threat to human beings (Ip et al. 2007; Jia et al. 2021). As a result, the role of estuaries in altering the fate of metals and, their flux to oceans needs better understanding. (Paalman et al. 1994; Powell et al. 1996; Fang and Wang 2022; Harmesa et al. 2022).

Furthermore, there are a variety of human interferences at the sites, as well as varying degrees of trace metal exposure. Population groups can be exposed to metals by intake, inhalation or dermal contact (Kim et al 2004; Bhattacharya et al. 2015). Since majority of the coastal community on the west coast of Karnataka are fishermen by occupation and spend most of their time in the water, we are only looking at dermal absorption for our research. Thus, quantifying the concentration of trace elements in the aquatic environment to control and mitigate the pollution of the ecosystem is very much essential.

With this overview, the present study is taken up in the southwest coast of India which is also thickly populated vis-à-vis the mainland. This region is located in the tropics, and the latter is home to 40% of the world population and covers 40% of the Earth’s surface area (Fedele et al., 2021). The approximate population in the study area is one million (Government of India, 2012). Limited studies exist on Indian tropics pertaining to metal behaviour and risk assessment, and this can be a good baseline study, reported for the first time. Fisheries is thriving in the study area with a net annual income of 80,000 USD per hectare (Ramachandra et., 2013). Fish is consumed by a large majority of the coastal population in the study area which can indirectly impact the health of the population by bioaccumulation (trace metals in this study). Thus, the outcome of the study will evaluate the impact of pollutants in the transition zone, which in future can be used to implement pollution prevention strategies by the policy and decision-makers. With this overview, an attempt is made to understand the processes and functioning of the estuaries. The objectives of this work are twofold: (i) to study the distribution pattern of dissolved trace metals along the salinity gradients and (ii) to evaluate the risk of trace metals on human health.

## Study area

Swarna, Sharavati and Kali, the three main river estuaries, debouching into the Arabian Sea on the southwest coast of India, were considered. Swarna, an important river of the Udupi district, serves as a primary source of drinking water for Udupi-Manipal towns. The river originates in Karkala taluk in the Western Ghats and flows for 80 km before joining the Arabian Sea. Similarly, the river Sharavati originates in Ambutheertha and spreads across two districts of Karnataka, Shimoga and Uttara Kannada. The river flows for 128 km and discharges 324 m^3^/sec annually to the Arabian Sea (Amrish et al., 2021). River Kali, the longest perennial river of coastal Karnataka (length184 km), has an annual discharge of 210 m^3^/sec to the Arabian Sea. Southwest monsoon (June–September) is the primary water source for these rivers, with 350 to 400 cm annual precipitation contributing to 80% of the annual discharge. Thus, because of the huge discharge, these estuaries remain freshwater dominated during the monsoon season. Post-monsoon and pre-monsoon accounts for ~ 15 and 5% of the total discharge. Semidiurnal tides, massive discharge, wind energy, sediment load and tidal currents, are the main characteristics of these southwest estuaries. These estuaries are river-dominated with a smaller residence time of water and vary from a few days to a few months (Duinker.,1986; Suja et al. 2017; Fernandes et al. 2012; Nasnodkar & Nayak 2015).

Marine ecosystems contribute to 63% of the ecosystem services than terrestrial ecosystems which accounts for only 38%. Among the various services offered by the ecosystem, the fishery sector serves as the major livelihood option of the estuarine-dependent communities in the coastal villages. The banks of the Swarna are densely populated and has one of the important ports (Malpe), a major fishing harbour of the Karnataka coast. Furthermore, the net income generated from the utilisation of fish resources from the Kali and Sharavati estuaries is 23.05 and 3.24 crores respectively. Also, Kali and Sharavati support agricultural production and accounts for about 23.53% and 37.43% of the total production of the district. Also, Kali serves as one of the major contributors to the revenue generated from the extraction of mangrove products with a net income of 5.7 million annually. Similarly, Sharavati has an income of 5.2 million annually. Apart from these, these estuaries also offer regulating services (Kumar and Kumar 2008; Ramachandra et al. 2013a, b).

## Materials and methods

**Fig. 1:**
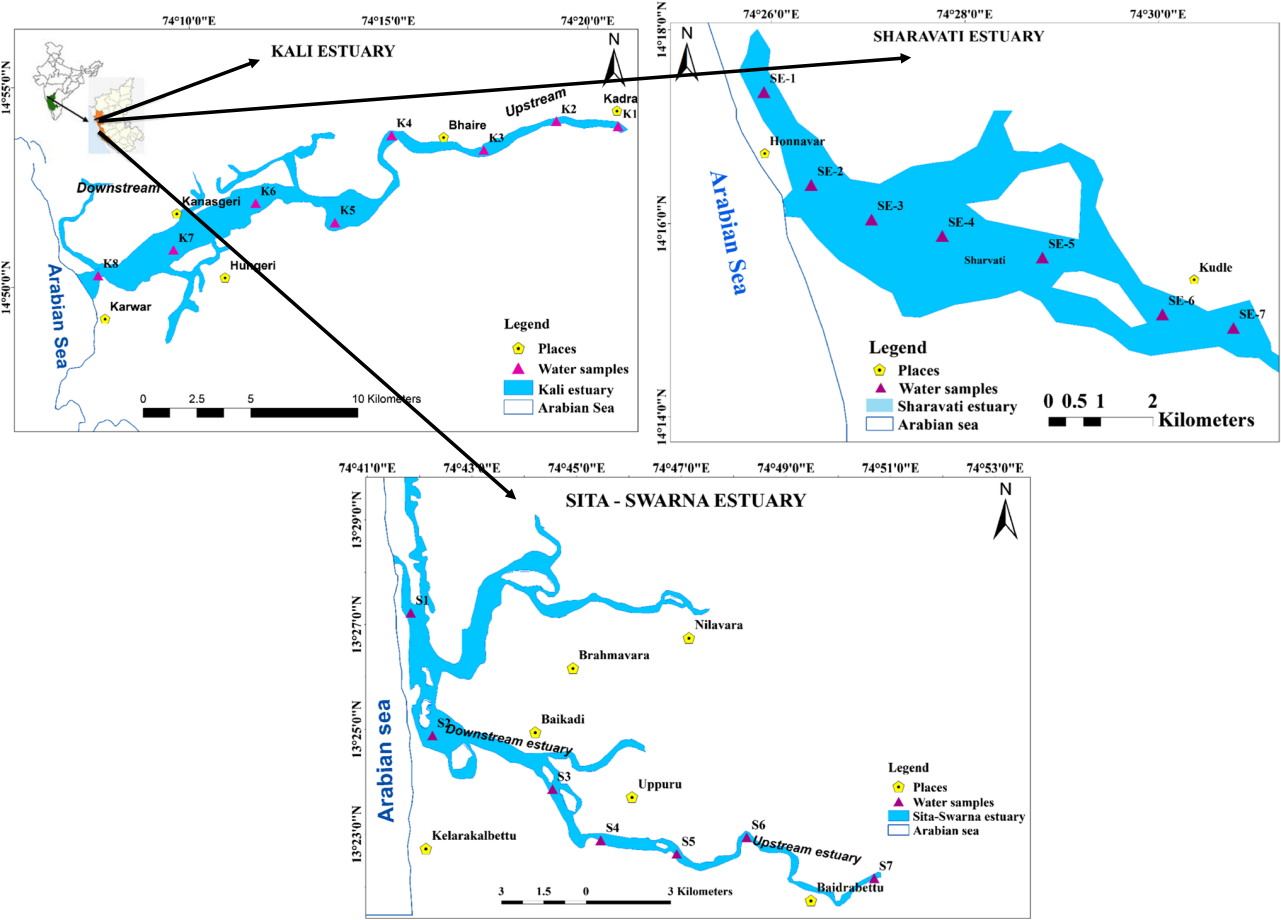
Map showing the sampling points along the salinity gradient of Sita-Swarna, Sharavati and Kali estuaries (Nishitha et al., 2021)

Surface seawater samples were collected along the salinity gradients of Sita-Swarna, Sharavati and Kali estuaries during the post-monsoon and pre-monsoon seasons using a speed boat. A pre-cleaned transparent polyethylene bucket was used to collect the water samples. Before the day of sampling, all the plastic bottles (polypropylene bottles; PP grade) were rinsed thrice with deionised water (18.2 mΩ cm^−1^ resistivity) prepared with Elga water purification system (UK) and kept for drying. As the water samples were collected for multiple measurements (nutrients, dissolved organic carbond, etc.), these bottles of 1-L capacity were not washed with acid. However, the PP bottles (250-ml capacity) used for trace metal collection were acid washed with 0.2 N Suprapur® nitric acid. The details of the sampling methods adopted can be found in the earlier work (Nishitha et al., 2021). Later in the field, at each location before collecting the sample, the bottles were rinsed thrice with the seawater. Physico-chemical parameters and salinity were measured immediately after the collection using HACH multi-parameter kit and salinity refractometer. The number of samples collected was not uniform because of varying salinities in short intervals. Six samples in Swarna river and seven samples each in Sharavati and Kali were collected. The sampling point coordinates were noted using the global positioning system (GPS) shown in Fig. 1. Finally, all the samples were carefully taken to the laboratory for further analysis.

Furthermore, the water samples for trace metal determination were filtered immediately using 0.22-${\varvec{\mu}}$ m pore-size Nuclepore filter paper. Immediately after the filtration, the water samples were transferred to a PP grade bottles which were soaked overnight in 0.2 N Suprapur® nitric acid. Furthermore, all the samples were acidified to pH < 2.0 using Suprapur® nitric acid (HNO_3_). Trace metals were determined using high-resolution inductively coupled mass spectrometer (HR-ICPMS) at the Physical Research Laboratory (PRL) Ahmedabad. The measurements were performed following the procedure similar to that reported by Hathorne et al. 2012. Online pre-concentration was performed using a commercially available SeaFAST system (Elemental Scientific Inc., Nebraska, USA).

The SeaFAST uses a resin with ethylenediaminetriacetic acid and iminodiacetic acid functional groups to pre-concentrate metals, whereas anions, as well as alkali metal and alkaline earth metal cations, are washed out. Thus, trace metals were absorbed by the chelating resin during pre-concentration. Later, these were eluted with 1.4 M HNO_3_ (prepared from Suprapur, Merck) and subsequently introduced to HR-ICP-MS. Calibration standards were prepared with diluted trace metal-free seawater. Trace metal-free seawater was prepared by passing it through the similar pre-concentration resin column used in the SeaFAST system to match the matrix of samples. Buffer solution (pH 6.0 ± 0.2) was prepared by mixing glacial acetic acid (CH_3_COOH) (Suprapur®, Merck) and 29% ammonium hydroxide (NH_4_OH) (Suprapur®, Merck) and used at a continuous flow to maintain the pH of the resin column during a pre-concentration step. Instrument drift correction was done by normalising the signals with that of indium internal standard. The repeatability of the samples was checked by analysing the duplicates and known concentration standards. One control standard was placed in the sample analysis sequence for every ten samples. The accuracy of the analytical procedure was evaluated by the measurement of certified estuarine water standard, SLEW-3 (National Research Council of Canada) and was within ± 5%. Table S1 shows the measured values of the seawater standards with the percentage recovery. Repeatability with the check standards was consistent and found within 10%.

A risk assessment study was undertaken to evaluate the impact of trace metals on the environment and human health. There are two distinct mechanisms by which humans encounter a risk of trace metals from water: (i) through the skin (dermal) and (ii) through water consumption. As a result, the investigated elements were compared to reference dosages and chronic daily intake (CDI). CDI_ingestion_ and CDI_dermal_ were estimated separately for the different age groups, i.e., adults and children, based on U.S. environmental protection agency (USEPA) standards. The following Eqs. 1 and 2 were adopted from USEPA (1996); (Mitra et al. 2018) to estimate the dose of exposure through dermal and ingestion routes.

1 $$\mathrm{CDI}\;_{\mathrm{ingestion}}=\frac{EC\;\mathrm x\;Ing\;R\;\mathrm x\;ED\;\mathrm x\;EF}{BW\;\mathrm x\;AT}$$

2 $${\mathrm{CDI}}_{\;\mathrm{dermal}}=\frac{EC\;\mathrm x\;SA\;\mathrm x\;AF\;\mathrm x\;ABSd\;\mathrm x\;ET\;\mathrm x\;EF\;\mathrm x\;ED\;\mathrm x\;CF}{BW\;\mathrm x\;AT}$$

*EC* is the measured metal concentration in seawater (mg/l), *IngR* is the average daily ingestion rate, (2.5 and 0.78 l/day: USEPA 1989) for adults and children respectively. *ED* represents exposure duration of life expectancy which is considered as 70 years for adults and 6 years for children respectively (USEPA 2002). *EF* is the frequency of exposure which is 365 days/year (USEPA 1989) as water is consumed every day. *BW* is body weight 70 and 15 kg for adults, and children respectively (USEPA 1991). *AT* is exposure time, taken as *ED* × 365 days (USEPA 1989). SA represents the skin area exposed which is (5700 cm^2^; USEPA 2011). *AF* is the factor of adherence which is taken as (0.07 mg cm^2^; USEPA 2011). *ABS*_*d*_ is the dermal absorption fraction (0.03; USEPA 2011). *ET* is the time of exposure (8 h/day; Bhattacharya et al. 2015), and *CF* is the conversion factor in kg/mg (10^−6^; USEPA 2002).

Furthermore, the hazard quotient for each metal is calculated using Eq. 3. A hazard quotient (HQ) is the ratio of a substance’s potential exposure to the level at which no adverse effects are expected. If the hazard quotient is less than one, no adverse health effects are anticipated as a result of exposure.

3 $$\mathrm{HQ}=\frac{\mathrm{CDI}}{RfD}$$

By adding all-metal hazard quotients, the potential human health risk is estimated. The index is known as Hazard Index (HI), and it is calculated using Eq. 4.

4 $$\mathrm{HI}=\sum\nolimits_{i=1}^{n}\mathrm{HQ}$$

The reference doses *RfD* is expressed as µg/kg/day and the values used are based on USEPA (2006, and 2010), except for Pb, a value derived from World Health Organization recommendation (WHO 2006) was used. The following *RfD*_ingestion_ and *RfD*_dermal_ values were used to calculate HQ for adults and children: For *RfD*_ingestion_, Fe-300, Al-1000, Mn-20, Cu-40, Zn-300, Ni-20, Cd-0.5, V-1, Cr-3, Co-0.3, As-0.3 and Pb-1.4; for RfD_dermal_, Fe-45, Al-200, Mn-0.8, Cu-12, Zn-60, Ni-5.4, Cd-0.005, V-0.01, Cr-0.015, Co-0.06, As-0.0123 and Pb-0.42. The values of HI and HQ (> 1) were considered as high risk for human health (Bhattacharya et al. 2015; Mitra et al. 2018).

In addition, the results were also compared with the seawater quality criteria (SWQC) prescribed by Karthikeyan et al. (2021) and USEPA, National Recommended Water Quality Criteria for aquatic life. SWQC was defined as the criterion maximum concentration (CMC), criterion continuous concentration (CCC) and predicted no effect concentration (PNEC) values. The standard values used for the comparison of the present study are listed in the (Table S2).

## Results

Table 1 shows the descriptive statistics of surface water samples collected along the salinity gradients in the Swarna, Sharavati and Kali estuaries. During the study period, salinity varied from 0.02 to 31.50 ppt during post-monsoon and from 2.00 to 36.50 ppt during pre-monsoon seasons. Similarly, pH showed an increasing trend towards the sea indicating the alkaline nature. Higher DO values were noted during the post-monsoon season in all the estuaries and comparatively lower values in the pre-monsoon season. The dissolved organic carbon (DOC) exhibited removal and desorption at different salinities. Detailed variations in salinity, pH and organic carbon in these estuaries are discussed in our recent study (Nishitha et al. 2021).

**Table 1:** Descriptive statistics of physico-chemical parameters and trace metals

Parameters	Swarna estuary				Swarna estuary			
	**Post-monsoon**				**Pre-monsoon**			
	Min	Max	Mean	SD	Min	Max	Mean	SD
Salinity (ppt)*	0.60	15.00	9.60	5.63	5.00	36.50	26.85	14.85
pH	7.85	8.44	8.23	0.23	7.36	8.23	7.92	0.49
DO (mg/l)	7.10	9.52	7.96	0.83	6.54	7.50	7.10	0.50
DOC (mg/l)	1.79	4.01	3.05	0.77	2.05	2.81	2.48	0.39
Mn	8.80	522.67	123.98	198.82	6.20	113.98	43.34	49.42
Fe	25.70	129.22	56.43	38.44	17.95	27.41	21.53	4.11
Co	0.36	1.58	0.71	0.46	0.39	1.93	0.93	0.71
Ni	4.96	13.37	8.92	3.36	5.72	9.98	8.37	2.01
Cu	6.01	11.43	8.90	2.47	6.40	14.90	9.16	3.91
Cd	0.20	0.60	0.41	0.14	0.46	0.57	0.51	0.05
Pb	0.41	1.13	0.59	0.27	0.10	1.10	0.58	0.52
Parameters	**Kali estuary**				**Kali estuary**			
	**Post-monsoon**				**Pre-monsoon**			
	Min	Max	Mean	SD	Min	Max	Mean	SD
Salinity (ppt)	0.02	11.08	4.56	4.55	2.00	31.60	16.21	12.38
pH	7.65	8.37	8.12	0.32	7.78	8.48	8.19	0.22
DO (mg/l)	7.22	8.19	7.68	0.35	6.43	8.31	7.35	0.67
DOC (mg/l)	0.86	1.46	1.19	0.23	1.52	3.56	2.08	0.70
Mn	4.39	16.08	8.98	3.86	3.34	704.53	168.39	285.83
Fe	7.08	32.47	18.15	9.48	7.30	48.09	19.37	13.92
Co	0.10	0.30	0.17	0.08	0.15	0.41	0.23	0.09
Ni	3.79	6.86	5.25	1.04	3.81	11.02	5.81	2.52
Cu	3.53	8.11	6.00	1.42	4.97	41.96	16.40	12.90
Cd	0.04	0.57	0.22	0.19	0.08	0.59	0.32	0.20
Pb	0.21	0.48	0.33	0.10	0.12	0.50	0.21	0.14
Parameters	**Sharavati estuary**				**Sharavati estuary**			
	**Post-monsoon**				**Pre-monsoon**			
	Min	Max	Mean	SD	Min	Max	Mean	SD
Salinity (ppt)	0.7	16.6	6.92	5.45	4.57	30.80	17.36	10.96
pH	7.60	8.23	8.05	0.23	7.72	8.17	8.04	0.21
DO (mg/l)	7.13	9.51	8.60	0.83	6.47	6.84	6.68	0.15
DOC (mg/l)	1.17	2.26	1.64	0.36	1.46	1.84	1.66	0.17
Mn	95.06	146.40	121.45	20.50	7.58	334.99	93.47	161.15
Fe	34.24	62.94	50.47	11.91	18.22	77.53	47.10	24.25
Co	0.11	0.45	0.30	0.14	0.12	0.22	0.17	0.04
Ni	3.68	11.32	6.02	2.85	4.97	6.17	5.54	0.56
Cu	7.51	127.69	31.61	47.15	14.67	40.58	27.03	13.49
Cd	0.06	0.23	0.13	0.07	0.40	0.68	0.51	0.13
Pb	0.13	0.21	0.16	0.03	0.10	0.19	0.15	0.04

^*^Trace metals in nmol/kg and salinity in parts per thousand (ppt)

Furthermore, dissolved trace metals analysed from Swarna, Sharavati and Kali estuaries showed significant spatiotemporal variations along the salinity gradients. Among all the metals studied, Mn had the highest values in the Kali estuary, compared to Swarna (< 0.75 times than Kali) and two times higher than Sharavati estuary. Swarna, on the other hand, had three times the Fe concentrations of Kali and 23 times the Fe concentrations of Sharavati estuaries. Similarly, metal Co showed four times higher values in Swarna than compared to Sharavati and Kali. Whereas metal Ni showed similar concentrations in all the three estuaries. However, Sharavati estuary showed higher Cu and Cd values, nearly 1.6 and 1.4 times greater than Swarna and Sharavati estuaries respectively. Similarly, Pb was found to be high in Swarna than compared to Sharavati and Kali estuaries Thus, Kali showed dominance for metal Mn and Swarna for metals Fe, Co and Pb. Sharavati on the other hand showed dominance for metals Cu and Cd.

Furthermore, the concentrations of Mn in the Swarna estuary ranged between 0.4 and 30.0 µg/l. In the Sharavati and Kali estuaries, it was 0.2 µg/l and 19.0 µg/l and 0.19 µg/l and 40.07 µg/l, respectively. Fe concentrations in the Swarna estuary ranged between 1.0 and 69 µg/l. Whereas, it ranged between 0.5 and 25 µg/l and 0.4 and 3.0 µg/l in the Sharavati and Kali estuaries, respectively. Furthermore, Co levels in Swarna estuary ranged from 0.02 to 0.1 µg/l. Sharavati and Kali, on the other hand, recorded the same values 0.01 to 0.03 µg/l and 0.01 to 0.03 µg/l. Similarly, the Ni levels in Swarna estuary ranged from 0.3 to 0.8 µg/l. Sharavati and Kali, on the other hand, recorded the same values 0.2 to 0.7 µg/l and 0.2 to 0.7 µg/l. Likewise, Cu concentrations in the Swarna estuary ranged between 0.4 and 1.0 µg/l. Whereas, it ranged between 0.3 and 8.4 µg/l and 0.2 and 3.0 µg/l in the Sharavati and Kali estuaries, respectively. Similarly, the concentrations of Cd in the Swarna estuary ranged between 0.02 and 0.07 µg/l. In the Sharavati and Kali estuaries, it was 0.01 µg/l and 0.08 µg/l and 0.01 µg/l and 0.07 µg/l, respectively, Pb concentrations in the Swarna estuary ranged between 0.02 and 0.2 µg/l. Whereas, it ranged between 0.02 and 0.05 µg/l and 0.03 and 0.1 µg/l in the Sharavati and Kali estuaries, respectively. Thus, irrespective of the estuary, trace metals showed significant spatial and temporal variations along the salinity gradient because of the mixing of fresh water with the seawater.

## Discussions

In order to identify the estuarine reactivity in modifying the fate of trace metals, a mixing graph approach was used. In these diagrams, the concentrations (including the end-member waters) were plotted against a conservative index of mixing, i.e., a component whose concentration is controlled only by physical mixing. Thus, TDL (theoretical dilution line), joining the two end members of the mixing series, was used as a base to understand the distribution and behaviour of the dissolved trace metals in the transition zone. For example, when the component participates in estuarine reactions, its concentrations will deviate from the TDL as a result of its addition to, or loss from, the dissolved state. These actions are referred to as non-conservative or reactive. If such a component is added to a mixture, the dissolved concentration data will be higher than the TDL and if removed from the solution, it will be below the TDL. Whereas, if a component remains non-reactive, its concentrations will plot exactly on the TDL. In such a case, it is considered as conservative or non-reactive.

In the present study, salinity was used as a conservative index and the dissolved trace metal concentrations were plotted as a function of salinity for both post-monsoon and pre-monsoon periods. The pattern of distribution of respective metals (Mn, Fe, Co, Ni, Cu, Cd and Pb) and their behaviour in the dissolved phase are discussed separately in the following section.

### Distribution of dissolved Mn and Fe

**Fig. 2:**
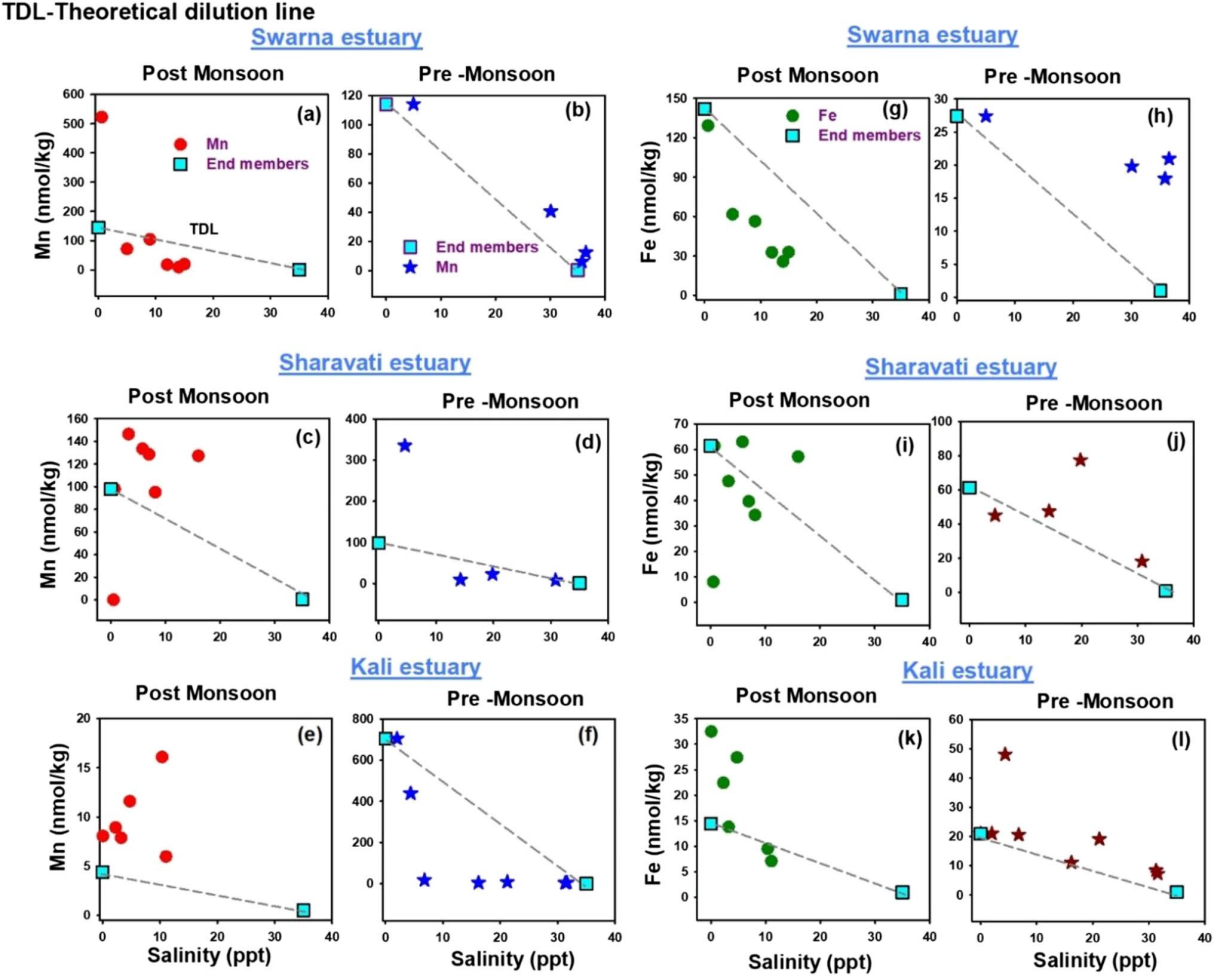
Spatiotemporal distribution of Mn and Fe along the salinity gradient of Swarna, Sharavati and Kali estuary (TDL: Theoretical dilution line)

The elements iron and manganese are essential in the aquatic geochemical processes because their oxides and hydroxides act as scavengers for various trace elements. The distribution of Mn in estuaries is variable with both gain and losses. Conservative behaviour is also reported in the literature (Dunker et al., 1986). Figure 2(a and b), (c and d) and (e and f) show the distribution of dissolved Mn along the salinity gradients of Swarna, Sharavati and Kali estuaries, respectively. Irrespective of the sampling periods, dissolved Mn showed non-conservative behaviour with higher concentrations in the low salinity regions. The enrichment of Mn at low salinity region was observed, which is mainly because of the fluvial source, i.e., mixing of fresh water and seawater causes changes in the ionic strength which finally results in the desorption of Mn from the suspended particulate matter. Also, Mn is a redox-sensitive element that can readily undergo transformation between the dissolved and the particulate phases in response to the changes in the physicochemical parameters. This is a commonly observed phenomenon in other estuarine systems (Li et al. 1984; Hatje et al. 2003; Shim et al. 2012; Joung and Shiller 2016). In the case of Swarna estuary, dissolved manganese showed a high concentration at low salinities and followed a decreasing trend towards the sea mouth. Similar behaviour was observed in the pre-monsoon period too.

Furthermore, dissolved Mn gradually increased towards higher salinity in the Kali estuary with a maximum value at 10 ppt. Their concentrations decreased towards the sea mouth. Desorption of Mn from the benthic sediments due to the microbial activity caused increased Mn in the mid salinity. Similar behaviour was reported by Joung and Shiller (2016). Sharavati showed the rapid removal of dissolved Mn in the mid salinities with non-conservative behaviour during the post-monsoon season. Thus, even though Mn is removed from the dissolved phase in many estuaries, it does appear to exhibit a global estuarine mixing pattern.

Furthermore, Fe is one of the most particle reactive elements, and the global behavioural pattern of Fe removal in estuaries has been reported by many workers (Windom et al. 1988, 1991, 1999; Beck et al. 2010). Fe showed non-conservative behaviour in all the three estuaries in the present study, irrespective of the sampling periods. Gradual removal of dissolved Fe along the salinity gradients of Swarna, Sharavati and Kali estuaries was observed, which is shown in Fig. 2(g and h), (i and j) and (k and l) respectively. Whereas, during the pre-monsoon season, rapid removal of Fe at the early stages of the estuarine mixing was observed in Swarna and Kali estuaries. This is consistent with the earlier findings in many estuaries. (Sholkovitz 1976; Boyle et al. 1977; Powell and Wilson-Finelli 2003; Shim et al. 2012). The non-conservative losses are mainly associated with the flocculation of colloidal materials in low salinity waters (Shim et al. 2012).

### Distribution of dissolved Co and Ni

**Fig. 3:**
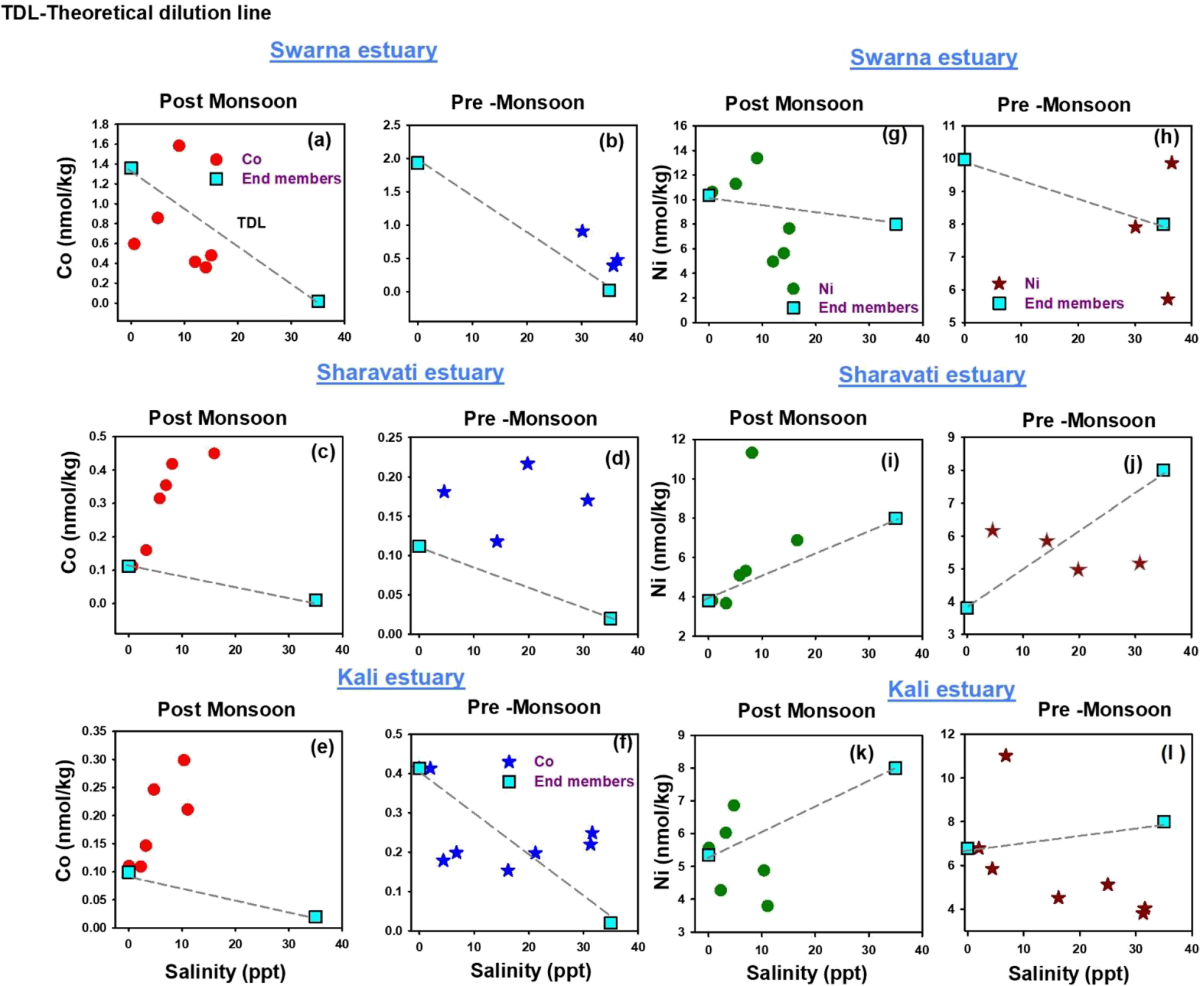
Spatiotemporal distribution of Co and Ni along the salinity gradient of Swarna, Sharavati and Kali estuary

Dissolved Co plotted as a function of salinity showed maximum concentrations in the mid salinities of Swarna (Fig. 3a and b) and Sharavati (Fig. 3c and d) estuaries during post-monsoon season. At the same time, the Kali estuary (Fig. 3e and f) showed a continuous increase in the Co concentration towards the sea mouth. The increase in the dissolved Co concentrations with the distance from the riverine end member in all the three estuaries suggests that, in addition to fluvial sources, some other sources also contribute to high levels of dissolved Co.

Co can be added to water by many sources like benthic remobilisation (Achterberg et al. 2003), desorption from the suspended particles (Li et al. 1984; Sanchez et al., 2004) and reduction of Mn oxides. The non-conservative excess of dissolved Co from other estuaries has been well documented in the literature (Martino et al. 2002; Sanchez et al.,2004). Furthermore, the distribution of dissolved Ni along the salinity gradients of Swarna, Sharavati and Kali estuaries is shown in Fig. 3(g and h), (i and j) and (k and l) respectively. During both seasons, dissolved Ni showed decreasing values close to the sea mouth, which could be because of biological uptake (Gaulier et al. 2021). Thus, dissolved Ni showed non-conservative behaviour or was unreactive during estuarine mixing. Both Co and Ni are particle reactive and are likely to undergo redox changes. Also, these elements form complexes with organic ligands and are subject to scavenging by particles of organic matter, oxides of Fe and Mn. (Velde et al. 2020).

### Distribution of dissolved Cu and Cd

**Fig. 4:**
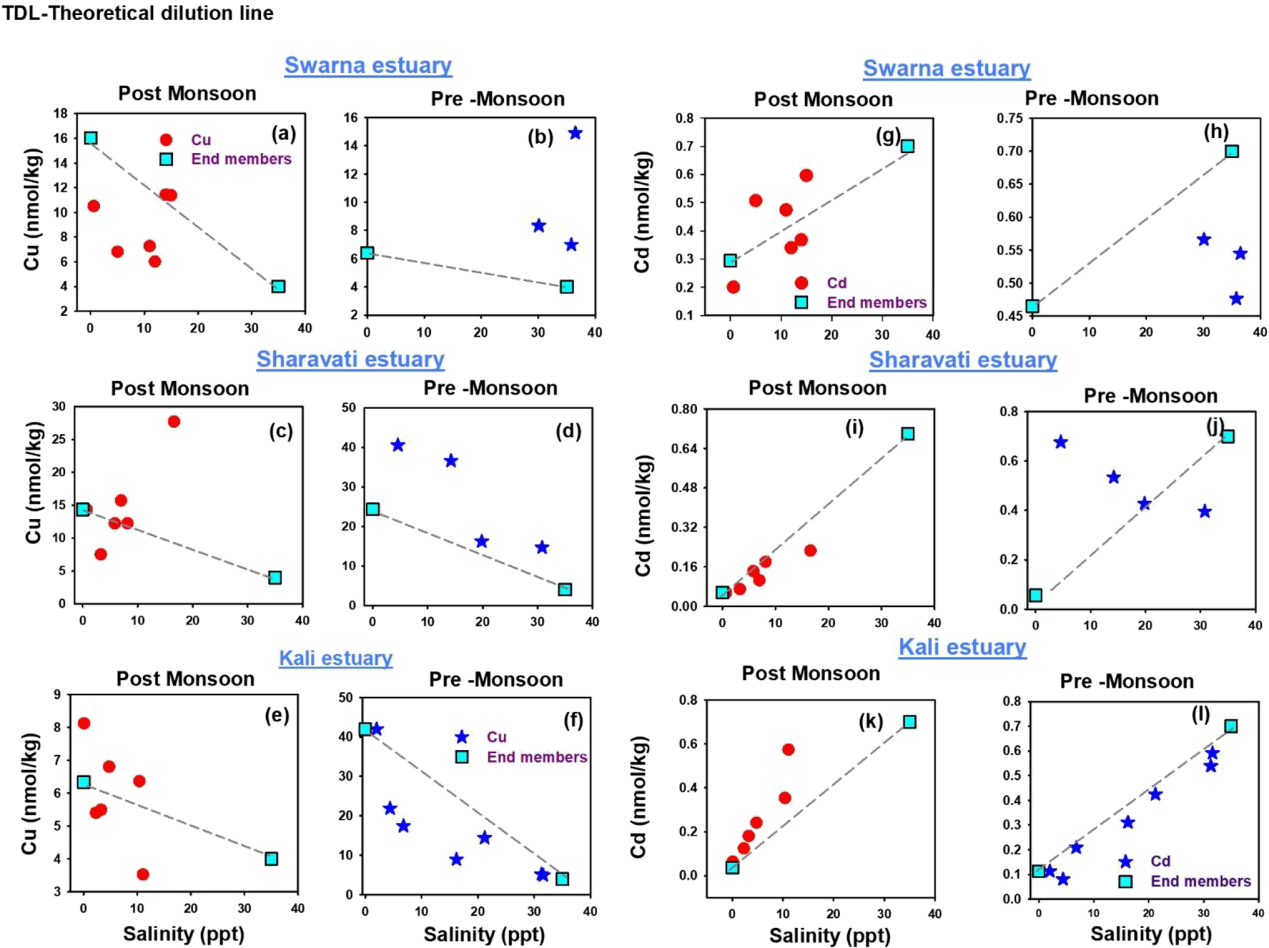
Spatiotemporal distribution of Cu and Cd along the salinity gradient of Swarna, Sharavati and Kali estuary

Dissolved Cu showed contrasting behaviour along the salinity gradients of Swarna (Fig. 4a and b), Sharavati (Fig. 4c and d) and Kali estuaries (Fig. 4e and f), respectively. During the post-monsoon season, removal of Cu at low salinity (between 0 and 4 ppt) and maxima at intermediate salinities was observed in all three estuaries studied. Similar behaviour was reported in Savannah and Ogeechee estuaries from the USA. Remobilisation from the bottom or resuspended sediment is likely to be the leading cause which is responsible for the increase in dissolved Cu concentrations at salinities more than 20 ppt (Sholkovitz et al. 1978; Windom et al. 1983). In contrast to the post-monsoon period, dissolved Cu showed conservative behaviour during pre-monsoon season with higher concentrations in the low salinity regions and followed a decreasing trend towards the sea mouth. This could be possible because of the resuspension of organic carbon from the bottom sediments (Lehman and Mills 1994). Thus, both conservative (Shiller and Boyle 1991; Shim et al. 2012) and non-conservative (Windom et al. 1983) behaviours of Cu have been observed in many large estuaries.

Figure 4 (g and h), (i and j) and (k and l) show the distribution of dissolved Cd along the salinity gradients of Swarna, Sharavati and Kali estuaries, respectively. In general, dissolved Cd showed an increasing tendency towards seaward in all the three estuaries during the post-monsoon period. The constant increase of Cd concentration is likely to be because of desorption from suspended particulate matter (Gaulier et al. 2021; Bingham et al. 1984). Regeneration from the degradation of organic matter could also influence the behaviour of dissolved cadmium in estuaries (Windom et al. 1991). In contrast, dissolved Cd showed rapid removal at low salinity in the Sharavati estuary (Fig. 4l) during the pre-monsoon season. Similar behaviour was reported by Jia et al. (2021).

### Distribution of dissolved Pb

**Fig. 5:**
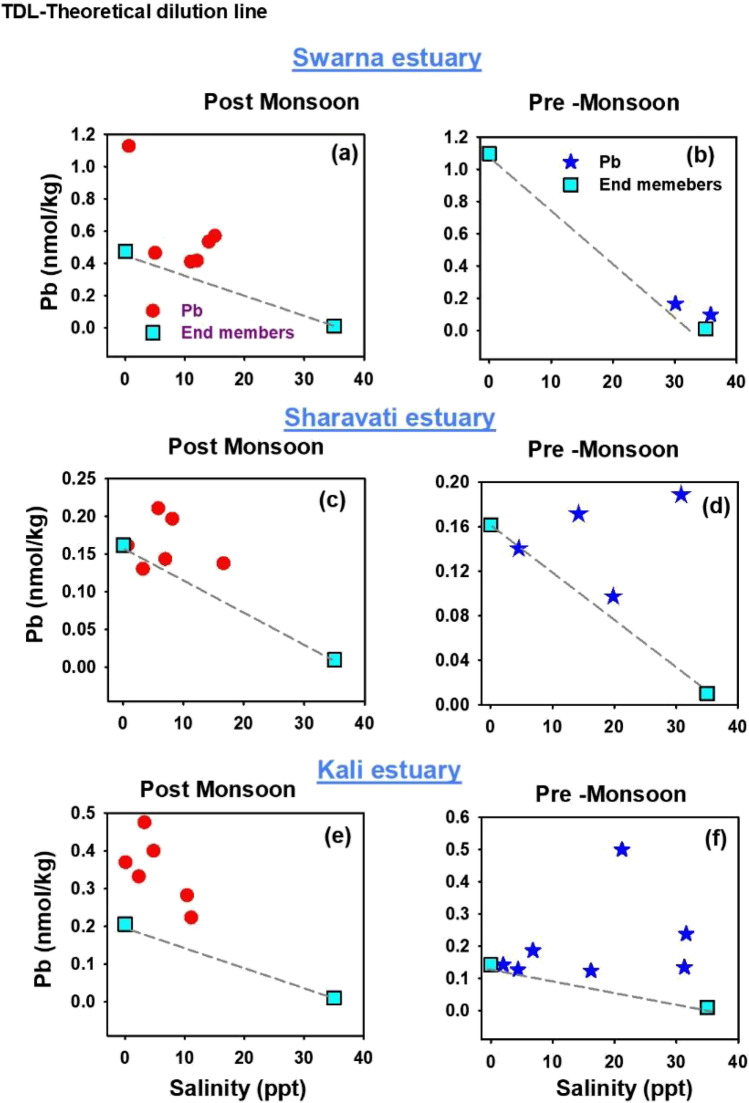
Spatiotemporal distribution of Pb along the salinity gradient of Swarna, Sharavati and Kali estuary

The distribution of dissolved Pb along the salinity gradients of Swarna, Sharavati and Kali estuaries is shown in Fig. 5(a and b), (c and d) and (e and f) respectively. Dissolved Pb plotted as a function of salinity showed non-conservative behaviour with gradual removal towards the sea. Many variations can be seen in the low salinity regions (i.e., close to the river). Hence, it can be ascertained that dissolved Pb concentrations seem to be influenced more by river water inputs than by salinity distribution in the estuary.

### Pearson correlation analysis

Pearson correlation analysis was performed using the software—Statistical Package for the Social Sciences (SPSS version 21) to understand the relationship between the dissolved trace metals and the different carrier phases (organic matter and Fe) in the studied estuaries. All the trace metals showed a strong relationship with physicochemical parameters such as salinity, pH and dissolved oxygen. From the analysis, it can be understood that, during the mixing of river water and saline water, the flocculation rates of Mn, Ni and Pb will increase as dissolved oxygen levels rise. On the other hand, Cu and Zn flocculation rates, will decrease. In addition, pH, salinity and hydrodynamic conditions will act as controlling factors causing sorption, desorption and precipitation of metals (Karbassi and Marefat., 2017) as, the particle—water reactions are time dependent and greatly influenced on the transport of trace metals (Millward., 1995). Thus, inter-elemental association was found to be weak indicating the different provenance and diverse behaviour during their transport in the transition zone. Overall, in all the estuaries, during the post-monsoon season, salinity showed strong relation with the metals thus being the major factor in controlling the precipitation and remobilisation of the metals in the estuarine environment. However, during the pre-monsoon season, most of the metals did not show a significant correlation with salinity in all three estuaries (Table 2).

**Table 2:** Pearson correlation analysis of trace metals in seawater during post-monsoon and pre-monsoon seasons

Parameters	Salinity	pH	DO	DOC	Mn	Fe	Co	Ni	Cu	Cd	Pb
**(a) Swarna–post-monsoon**											
Salinity	1										
pH	0.572	1									
DO	− 0.503	0.273	1								
DOC	− 0.253	− 0.67	− 0.41	1							
Mn	− .840*	− 0.177	.870*	− 0.024	1						
Fe	0.045	− 0.349	− 0.079	0.051	0.032	1					
Co	− 0.148	− 0.654	− 0.208	0.325	0.055	.935**	1				
Ni	− 0.537	− .835*	0.071	0.498	0.419	0.693	.860*	1			
Cu	0.15	0.44	0.566	− 0.36	0.221	− 0.316	− 0.443	− 0.228	1		
Cd	0.551	− 0.268	− 0.746	0.585	− 0.695	0.159	0.269	0.144	− 0.05	1	
Pb	− 0.69	0.058	.935**	− 0.125	.929**	− 0.247	− 0.258	0.156	0.515	− 0.657	1
(b) Swarna-pre-monsoon											
Salinity	1										
pH	.999*	1									
DO	− 0.618	− 0.656	1								
DOC	− 0.33	− 0.283	− 0.539	1							
Mn	− 0.963	− 0.975	0.807	0.063	1						
Fe	− 0.037	− 0.085	0.808	− 0.931	0.305	1					
Co	− 0.966	− 0.977	0.801	0.073	.999**	0.295	1				
Ni	0.065	0.017	0.744	− 0.964	0.206	0.995	0.196	1			
Cu	0.448	0.404	0.426	− 0.992	− 0.19	0.877	− 0.2	0.922	1		
Cd	− 0.608	− 0.646	.999**	− 0.549	0.8	0.816	0.794	0.753	0.438	1	
Pb	0.523	0.481	0.346	− 0.977	− 0.274	0.832	− 0.284	0.884	0.996	0.358	1
**(c) Kali-post-monsoon**											
Salinity	1										
pH	0.746	1									
DO	0.219	0.548	1								
DOC	0.675	0.75	0.054	1							
Mn	0.464	0.536	.811*	0.282	1						
Fe	− 0.664	− 0.404	− 0.094	− 0.307	0.021	1					
Co	.838*	0.726	0.549	0.53	.772*	− 0.361	1				
Ni	− 0.422	− 0.041	0.427	− 0.398	0.181	0.519	0.062	1			
Cu	− 0.589	− 0.541	0.224	− 0.649	0.28	0.747	− 0.143	0.636	1		
Cd	.954**	0.7	− 0.021	0.694	0.223	− 0.624	0.704	− 0.456	− 0.702	1	
Pb	− 0.289	0.275	0.512	− 0.22	0.239	0.48	− 0.048	0.652	0.326	− 0.291	1
**(d) Kali-pre-monsoon**											
Salinity	1										
pH	0.273	1									
DO	− 0.273	.782*	1								
DOC	− 0.496	− 0.687	− 0.16	1							
Mn	− 0.715	− .860*	− 0.424	0.722	1						
Fe	− 0.702	− 0.237	0.041	0.03	0.57	1					
Co	− 0.27	− .829*	− 0.563	.793*	0.714	− 0.095	1				
Ni	− 0.675	0.234	0.522	0.077	0.174	0.299	0.104	1			
Cu	− .826*	− 0.692	− 0.134	.832*	.917**	0.485	0.735	0.434			
Cd	.987**	0.292	− 0.237	− 0.449	− 0.735	− .769*	− 0.21	− 0.593	− .793*	1	
Pb	0.289	0.199	0.18	0.009	− 0.343	− 0.124	− 0.127	− 0.15	− 0.155	0.358	1
(e) Sharavati-post-monsoon											
Salinity	1										
pH	.420	1									
DO	− .623	.282	1								
DOC	− .042	− .253	.239	1							
Mn	.120	.220	.170	.268	1						
Fe	.109	.407	− .156	− .533	− .649	1					
Co	.871^*^	.746	− .286	− .170	− .053	.409	1				
Ni	.491	.573	− .311	− .477	− .539	.911^*^	.740	1			
Cu	.874^*^	− .064	− .792	.145	.110	− .190	.543	.154	1		
Cd	.925^**^	.515	− .524	.018	− .081	.385	.919^**^	.704	.718	1	
Pb	− .143	.335	.436	.358	− .408	.523	.202	.436	− .369	.221	1
(f) Sharavati-pre-monsoon											
Salinity	1										
pH	0.832	1									
DO	− 0.496	− 0.726	1								
DOC	0.745	0.365	− 0.429	1							
Mn	− 0.784	− .994**	0.691	− 0.267	1						
Fe	− 0.363	0.017	− 0.628	− 0.179	− 0.017	1					
Co	0.106	− 0.093	− 0.486	0.684	0.185	0.463	1				
Ni	− 0.829	− 0.788	0.861	− 0.793	0.722	− 0.19	− 0.528	1			
Cu	− 0.894	− 0.734	0.729	− 0.891	0.658	0.005	− 0.522	.976*	1		
Cd	− 0.947	− 0.924	0.746	− 0.693	0.879	0.049	− 0.216	0.939	0.935	1	
Pb	0.339	0.156	0.562	− 0.086	− 0.192	− 0.923	− 0.748	0.243	0.103	− 0.075	1

^**^Correlation is significant at the 0.01 level (2-tailed)

^*^Correlation is significant at the 0.05 level (2-tailed)

The “Bold” values indicate the statistically significant values at (p <0.05 and <0.01)

### Risk assessment

Human health risk assessment considers three different exposure pathways such as ingestion, inhalation and dermal absorption. In our study, we have focused on dermal absorption because that is the most probable mode that can affect humans in the estuarine environment of the study area (Kim et al 2004; Kumar et al. 2020). Health risk by ingestion or inhalation is minimal because estuarine water is brackish and not potable. Exceptions can be those people that are involved in various water sport activities such as divers, surfers and swimmers. Accidental, direct ingestion of seawater of approximately 50 ml is possible for these personnel during recreational activities (WHO 2003) which is considered negligible and hence not discussed further. Dermal exposure is the most probable especially for the fishermen who spend approximately 8 to 10 h per day in the estuary/nearshore which we think is similar to the ones reported in the East Coast of India (Bhattacharya et al. 2015).

**Fig. 6:**
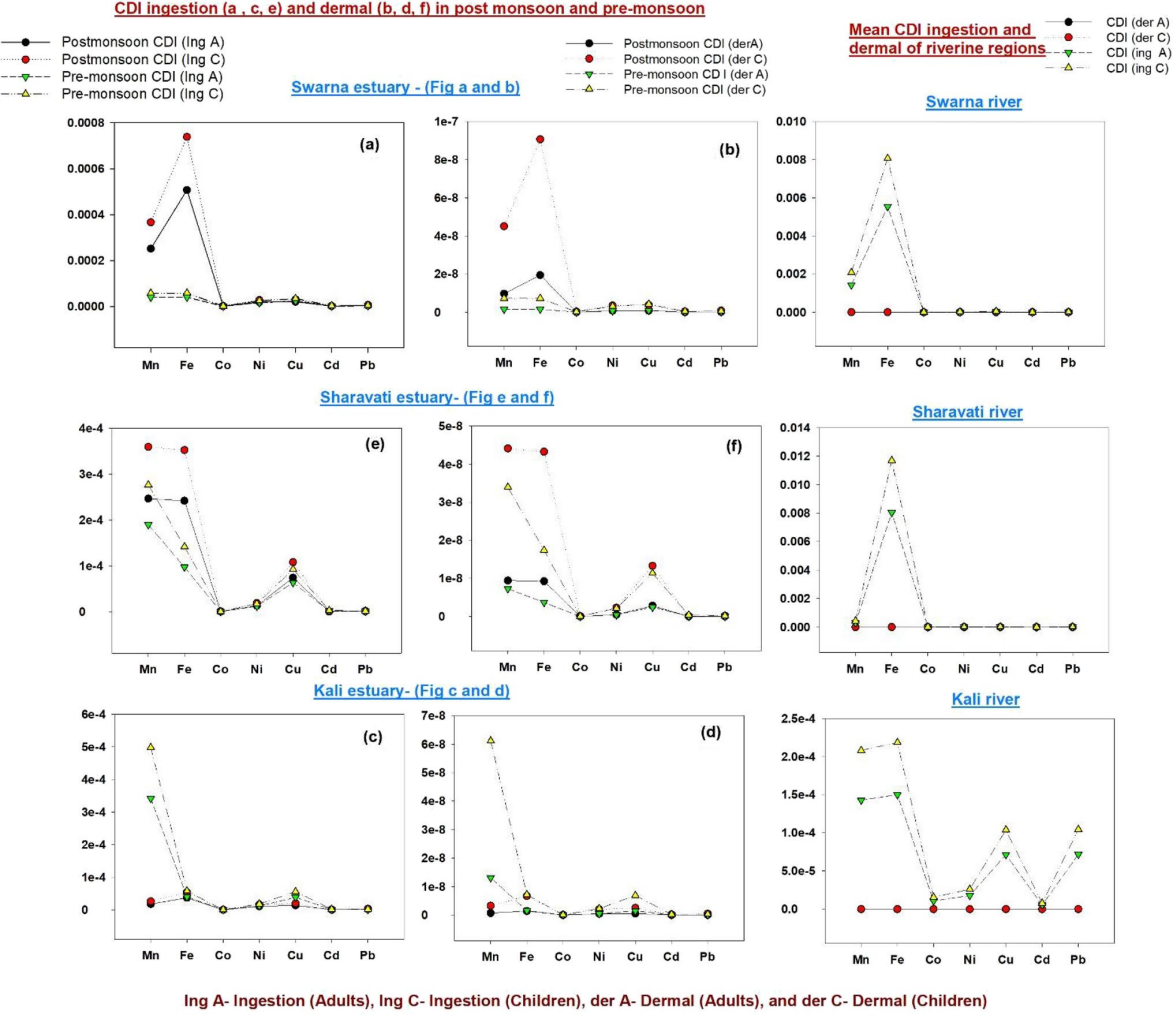
CDI ingestion and dermal rate between two population groups (adult and children) during post-monsoon and pre-monsoon seasons

Chronic daily intake through the dermal pathway of the local population during post-monsoon and pre-monsoon seasons was assessed (Table 3; Fig. 6). All the three estuaries, Swarna, Sharavati and Kali showed a similar pattern of exposure between pre-monsoon and post-monsoon seasons in adult and children groups with respect to the elements Co, Ni, Cu, Cd and Pb. On the other hand, exposure rates of Mn and Fe were much higher than other elements in all three estuaries. Deposits of Mn and Fe ore are reported from the Western Ghats which could be the probable sources. The risk assessment estimated at the river mouths (Table S3) (freshwater end-member) showed comparatively higher CDI values through ingestion pathway (Fig. 6) in both the population groups.

**Table 3:** Average values of CDI_dermal_ and HQ_dermal_ for adults and children’s from Swarna, Sharavati and Kali estuaries

			Adult		Children	
Swarna estuary-post-monsoon	Elements	Conc (mg/l)	CDI (dermal)	HQ (dermal)	CDI (dermal)	HQ (dermal)
	Mn	0.00705	9.64 × E^−09^	1.20 × E^−08^	4.50 × E^−08^	5.62 × E^−08^
	Fe	0.01420	1.94 × E^−08^	4.31 × E^−10^	9.06 × E^−08^	2.01 × E^−09^
	Co	0.00004	5.96 × E^−11^	9.93 × E^−10^	2.78 × E^−10^	4.63 × E^−09^
	Ni	0.00054	7.40 × E^−10^	1.37 × E^−10^	3.45 × E^−09^	6.40 × E^−10^
	Cu	0.00059	8.01 × E^−10^	6.67 × E^−11^	3.73 × E^−09^	3.11 × E^−10^
	Cd	0.00005	6.59 × E^−11^	1.31 × E^−08^	3.07 × E^−10^	6.15 × E^−08^
	Pb	0.00013	1.72 × E^−10^	4.09 × E^−10^	8.03 × E^−10^	1.91 × E^−09^
Swarna estuary-pre-monsoon	Mn	0.00113	1.54 × E^−09^	1.92 × E^−09^	7.18 × E^−09^	8.98 × E^−09^
	Fe	0.00113	1.54 × E^−09^	3.43 × E^−11^	7.22 × E^−09^	1.60 × E^−10^
	Co	0.00004	4.94 × E^−11^	8.24 × E^−10^	2.30 × E^−10^	3.84 × E^−09^
	Ni	0.00048	6.51 × E^−10^	1.20 × E^−−10^	3.03 × E^−09^	5.62 × E^−10^
	Cu	0.00066	9.07 × E^−10^	7.55 × E^−11^	4.23 × E^−09^	3.52 × E^−10^
	Cd	0.00006	8.42 × E^−11^	1.68 × E^−08^	3.92 × E^−10^	7.85 × E^−08^
	Pb	0.00009	1.18 × E^−10^	2.80 × E^−10^	5.50 × E^−10^	1.31 × E^−09^
Kali estuary-post-monsoon	Mn	0.00051	6.99 × E^−10^	8.73 × E^−10^	3.26 × E^−09^	4.07 × E^−09^
	Fe	0.00105	1.43 × E^−09^	3.18 × E^−11^	6.69 × E^−09^	1.48 × E^−10^
	Co	0.00001	1.45 × E^−11^	2.42 × E^−10^	6.78 × E^−11^	1.13 × E^−09^
	Ni	0.00032	4.35 × E^−10^	8.07 × E^−11^	2.03 × E^−09^	3.76 × E^−10^
	Cu	0.00039	5.40 × E^−10^	4.50 × E^−11^	2.52 × E^−09^	2.10 × E^−10^
	Cd	0.00003	3.57 × E^−11^	7.15 × E^−09^	1.66 × E^−10^	3.33 × E^−08^
	Pb	0.00007	9.59 × E^−11^	2.28 × E^−10^	4.47 × E^−10^	1.06 × E^−09^
Kali estuary-pre-monsoon	Mn	0.00958	1.31 × E^−08^	1.63 × E^−08^	6.11 × E^−08^	7.64 × E^−08^
	Fe	0.00112	1.53 × E^−09^	3.40 × E^−11^	7.14 × E^−09^	1.58 × E^−10^
	Co	0.00001	1.91 × E^−11^	3.19 × E^−10^	8.95 × E^−11^	1.49 × E^−09^
	Ni	0.00035	4.82 × E^−10^	8.93 × E^−11^	2.25 × E^−09^	4.17 × E^−10^
	Cu	0.00108	1.47 × E^−09^	1.23 × E^−10^	6.88 × E^−09^	5.74 × E^−10^
	Cd	0.00004	5.15 × E^−11^	1.030 × E^−08^	2.40 × E^−10^	4.80 × E^−08^
	Pb	0.00004	6.07 × E^−11^	1.44 × E^−10^	2.83 × E^−10^	6.75 × E^−10^
Sharavati estuary-post-monsoon	Mn	0.00691	9.44 × E^−09^	1.18 × E^−08^	4.40 × E^−08^	5.51 × E^−08^
	Fe	0.00677	9.26 × E^−09^	2.05 × E^−10^	4.32 × E^−08^	9.60 × E^−10^
	Co	0.00002	2.51 × E^−11^	4.19 × E^−10^	1.17 × E^−10^	1.95 × E^−09^
	Ni	0.00037	4.99 × E^−10^	9.25 × E^−11^	2.33 × E^−09^	4.32 × E^−10^
	Cu	0.00208	2.84 × E^−09^	2.37 × E^−10^	1.32 × E^−08^	1.10 × E^−09^
	Cd	0.00002	2.06 × E^−11^	4.13 × E^−09^	9.64 × E^−11^	1.92 × E^−08^
	Pb	0.00004	4.79 × E^−11^	1.14 × E^−10^	2.23 × E^−10^	5.33 × E^−10^
Sharavati estuary-pre-monsoon	Mn	0.00532	7.27 × E^−09^	9.09 × E^−09^	3.39 × E^−08^	4.24 × E^−08^
	Fe	0.00272	3.72 × E^−09^	8.27 × E^−11^	1.73 × E^−08^	3.86 × E^−10^
	Co	0.00001	1.43 × E^−11^	2.38 × E^−10^	6.67 × E^−11^	1.11 × E^−09^
	Ni	0.00034	4.60 × E^−10^	8.52 × E^−11^	2.14 × E^−09^	3.97 × E^−10^
	Cu	0.00178	2.43 × E^−09^	2.02 × E^−10^	1.13 × E^−08^	9.46 × E^−10^
	Cd	0.00006	8.09 × E^−11^	1.61 × E^−08^	3.77 × E^−10^	7.55 × E^−08^
	Pb	0.00003	4.38 × E^−11^	1.04 × E^−10^	2.04 × E^−10^	4.87 × E^−10^

The maximum CDI values through ingestion pathway at the river mouth for children were found to be 8.06 × 10^−3^, 11.7 × 10 × ^−3^ and 0.21 × 10^−3^ in Swarna, Sharavati and Kali respectively. Similarly, at the same time, for adults, it was found to be 5.54 × 10^−3^, 8.04 × 10^−3^ and 0.15 × 10^−3^ in Swarna, Sharavati and Kali rivers respectively. In contrast, the maximum CDI values through ingestion pathway at the estuary mouth, for children were found to be 7.97 × 10^−4^, 6.36 × 10^−4^ and 5.53 × 10^−4^ in Swarna, Sharavati and Kali estuaries respectively. Similarly, for adults, it was found to be 5.48 × 10^−4^, 4.37 × 10^−4^ and 3.80 × 10^−4^ in Swarna, Sharavati and Kali estuaries respectively. The maximum CDI values were calculated by taking the average of CDI values from both the seasons. Furthermore, the Hazard Index through ingestion mode was estimated. The HI_ingestion_ values for children were found to be 1.60 × 10^−4^ in Swarna river, 0.86 × 10^−4^ in Sharavati river and 1.57 × 10^−4^ in Kali river. Similarly, the HI_ingestion_ values for adults were found to be 1.10 × 10^−4^ in Swarna river, 0.59 × 10^−4^ in Sharavati river and 1.08 × 10^−4^ in Kali river.

However, HI_ingestion_ values for children were found to be 4.02 × 10^−5^ and 2.10 × 10^−5^ in Swarna estuary, 2.88 × 10^−5^ and 2.66 × 10^−5^ in Sharavati estuary and 1.00 × 10^−5^ and 3.54 × 10^−5^ in Kali estuary during the post-monsoon and pre-monsoon seasons respectively. Similarly, the HI_ingestion_ values for adults were found to be 2.76 × 10^−5^ and 1.44 × 10^−5^ in Swarna estuary, 1.98 × 10^−5^ and 1.82 × 10^−5^ in Sharavati estuary and 0.68 × 10^−5^ and 2.43 × 10^−5^ in Kali estuary during the post-monsoon and pre-monsoon seasons respectively. Thus, from analysis, among the river mouth and the estuary, higher values were registered at the river mouth rather than the estuary mouth. This could be due to the discharge of river-transported industrial, agricultural activities and municipal wastes (Fernandes and Nayak 2015; Ramachandra et al., 2012). Kali river water end-member showed higher CDI for Ni, Cu and Pb through ingestion pathway for both the population groups. This could be from the anthropogenic discharges in the upstream of the river as discussed in our recent study (Arun et al 2022).

In contrast, the HI_dermal_ values for children were found to be 1.27 × 10^−7^ and 0.93 × 10^−7^ in Swarna estuary, 0.8 × 10^−7^ and 1.21 × 10^−7^ in Sharavati estuary and 0.40 × 10^−7^ and 1.27 × 10^−7^ in Kali estuary during the post-monsoon and pre-monsoon seasons respectively. Similarly, the HI_dermal_ values for adults were found to be 2.72 × 10^−8^ and 2.01 × 10^−8^ in Swarna estuary, 1.70 × 10^−8^ and 2.59 × 10^−8^ in Sharavati estuary and 0.87 × 10^−8^ and 2.73 × 10^−8^ in Kali estuary during the post-monsoon and pre-monsoon seasons respectively. As a result of the combined results, all the estuaries and the respective rivers are below the risk limit (HI > 1) for both population groups. Thus, all the estuaries showed negligible HI and HQ values because of the low concentration of trace metals. The values registered in this study are consistent with those previously studied elsewhere in the world. Mitra et al. (2018) reported the HI_dermal_ values for adults and children as 3.1 × 10^−7^ and 14.4 × 10^−7^ in Ganga estuary. A similar result was reported by Wu et al. (2009) for Yangtze river, China where HI_dermal_ values ranged between 2.21 × 10^−9^ and 8.45 × 10^−5^*.* However, the hazard quotient values estimated for human health through the dermal pathway revealed that children are more vulnerable to risk compared to adults. The current finding determined that there may be little chances of contamination through ingestion and dermal exposure routes and that there may be a negligible health risk for humans when it comes to surface water intake and absorption from the study area. In addition, the present quality of seawater was evaluated in terms of toxic metal concentration.

In addition, the results were compared with the seawater quality criteria (SWQC) proposed by Karthikeyan et al. (2021) and USEPA (2007, 2016). The concentrations of the dissolved Mn in different seasons in the Swarna, Sharavati and Kali estuaries ranged from 0.19 to 40.0 µg/l which were less than the threshold set by USEPA (1993) (50 µg/l). Likewise, the concentrations of the dissolved Ni in different seasons in the Swarna, Sharavati and Kali estuaries ranged from 0.2 to 0.8 µg/l which were less than the threshold set by USEPA (1995) (74 µg/l). Furthermore, the concentrations of the dissolved Cu in different seasons in the Swarna and Kali estuaries ranged from 0.2 to 1.0 µg/l which were less than the threshold set by USEPA (2007) (4.8 µg/l). Whereas, Sharavati estuary showed higher value (8.48 µg/l) than the threshold limit. Similarly, the concentrations of the dissolved Cd in different seasons in the Swarna, Sharavati and Kali estuaries ranged from 0.01 to 0.08 µg/l which were less than the threshold set by USEPA (2016) (33 µg/l) and Karthikeyan et al (2021) (1.7 µg/l). Besides, the concentrations of the dissolved Pb in different seasons in the Swarna, Sharavati and Kali estuaries ranged from 0.02 to 0.24 µg/l which were less than the threshold set by USEPA (1984) (210 µg/l) and Karthikeyan et al (2021) (17 µg/l). Thus, except dissolved Cu in Sharavati estuary, rest of the dissolved trace metals analysed occur within safe limits in all three estuaries.

## Conclusions

The present study focuses on the spatio-temporal behaviour of dissolved trace metals along the salinity gradients of three tropical estuaries Swarna, Sharavati and Kali. From the investigations, it can be surmised that in all the estuaries, the dissolved elements Mn, Fe, Ni, Cd and Co showed non-conservative behaviour along the salinity gradients. In contrast, dissolved Cu showed conservative and non-conservative behaviour during pre-monsoon and post-monsoon seasons, respectively. The conservative behaviour of Cu during the pre-monsoon season could be because of the complexation of Cu with resuspended organic carbon. Furthermore, the hazard quotient values estimated for human health through the dermal pathway showed higher values for children group compared to the adult groups. This reveals that children are more vulnerable to risk compared to adults. In addition, the Hazard Index values for all the metals fall below the risk limit, implying that the present quality of the estuarine water would not cause any adverse dermal exposure to humans. Hence, present study serves as a baseline data which can help monitor the quality of Swarna, Sharavati and Kali estuaries and associated Hazard Index in the future. This will also assist decision-makers in determining those areas that have to be considered for coastal water management plans.

## Supplementary Information

Below is the link to the electronic supplementary material.

Supplementary file1 (DOCX 26 KB)

## Data Availability

Not applicable.
