# Supplementary material for: Risk assessment and spatio-temporal distribution of dissolved trace metals in Swarna, Sharavati and Kali estuaries, South-West Coast of India
Source: Environ Sci Pollut Res Int. 2022 Sep 6;30(4):9914–31. doi: 10.1007/s11356-022-22812-4 (PMC9898361; doi:10.1007/s11356-022-22812-4)
Supplement: Supplementary file 1 — Supplementary file1 (DOCX 26 KB) [file 11356_2022_22812_MOESM1_ESM.docx]

**Risk assessment and Spatio-temporal distribution of dissolved trace metals in Swarna, Sharavati and Kali estuaries, South-West Coast of India.**

D’Souza Nishitha ^a^, Athiyarath Krishnan Sudheer ^b^, Kumar Arun ^a^, Vadakkeveedu Narayan Amrish ^a^, Gaddam Mahesh ^b^, Harikripa Narayana Udayashankar ^a^, Keshava Balakrishna ^a *^

^a^ Department of Civil Engineering, Manipal Institute of Technology, Manipal Academy of Higher Education, Manipal 576 104, India

^b^ Geosciences division, Physical Research Laboratory, Navrangpura, Ahmedabad 380009, India

*Corresponding author: [k.balakrishna@manipal.edu](mailto:k.balakrishna@manipal.edu)

*Supplementary material*

Table S1: Analysis of the results and certified values of SLEW 3 for quality assurance

| **Trace metals (ppb)** | **Certified value** | **Measured value** | **Uncertainty (%)** |
| --- | --- | --- | --- |
| Mn | 1.61 | 1.52 | 94.1 |
| Fe | 0.568 | 0.63 | 90.2 |
| Co | 0.042 | 0.04 | 95.0 |
| Ni | 1.23 | 1.13 | 91.2 |
| Cu | 1.55 | 1.48 | 95.3 |
| Cd | 0.048 | 0.052 | 92.3 |
| Pb | 0.009 | 0.009 | 100.0 |

Table S2: Sea water Quality standards based on USEPA and Karthikeyan et al (2021)

| **Pollutants** | **Saltwater** | | | **Publication** | **Proposed** |
| --- | --- | --- | --- | --- | --- |
|  | **CMC (Acute)** | **CCC (Chronic)** | **PNEC** |  |  |
|  | **(µg/l)** | | |  |  |
| **Cadmium (Cd)** | 33 | 7.9 |  | 2016 | **USEPA**  National Recommended Water Quality Criteria - Aquatic Life Criteria Table |
|  | 1.7 | 1.1 | 0.92 |  | Karthikeyan et al (2021) |
| **Lead (Pb)** | 210 | 8.1 |  | 1984 | USEPA |
|  | 17 | 5.9 | 4.3 |  | Karthikeyan et al (2021) |
| **Nickel (Ni)** | 74 | 8.2 |  | 1995 | USEPA |
| **Copper (Cu)** | 4.8 | 3.1 |  | 2007 |  |

Table S3: Average values of CDI _dermal_ CDI _ingestion,_ and HQ _ingestion,_ HQ_dermal_ for adults and children’s from Swarna, Sharavati and Kali rivers

|  | | | **Adult** | | | | **Children** | | | |
| --- | --- | --- | --- | --- | --- | --- | --- | --- | --- | --- |
|  | **Elements** | **Conc (mg/l)** | **CDI (dermal)** | **CDI (ingestion)** | **HQ (dermal)** | **HQ (ingestion)** | **CDI (dermal)** | **CDI (ingestion)** | **HQ (dermal)** | **HQ (ingestion)** |
| **Swarna** | Mn | 0.04 | 5.47 x E^-08^ | 1.43 x E^-03^ | 6.84 x E^-08^ | 7.14 x E^-05^ | 2.55 x E^-07^ | 2.08 x E^-03^ | 3.19 x E^-07^ | 1.04 x E^-04^ |
|  | Fe | 0.155 | 2.12 x E^-07^ | 5.54 x E^-03^ | 4.71 x E^-09^ | 1.85 x E^-05^ | 9.90 x E^-07^ | 8.06 x E^-03^ | 2.20 x E^-08^ | 2.69 x E^-05^ |
|  | Co | 0.0001 | 1.73 x E^-10^ | 4.51 x E^-06^ | 2.88 x E^-09^ | 1.50 x E^-05^ | 8.05 x E^-10^ | 6.56 x E^-06^ | 1.34 x E^-08^ | 2.19 x E^-05^ |
|  | Ni | 0.0003 | 3.80 x E^-10^ | 9.91 x E^-06^ | 7.03 x E^-11^ | 4.96 x E^-07^ | 1.77 x E^-09^ | 1.44 x E^-05^ | 3.28 x E^-10^ | 7.22 x E^-07^ |
|  | Cu | 0.0007 | 9.94 x E^-10^ | 2.60 x E^-05^ | 8.28 x E^-11^ | 6.49 x E^-07^ | 4.64 x E^-09^ | 3.78 x E^-05^ | 3.87 x E^-10^ | 9.45 x E^-07^ |
|  | Cd | - | 5.47 x E^-13^ | 1.43 x E^-08^ | 1.09 x E^-09^ | 2.86 x E^-08^ | 2.55 x E^-12^ | 2.08 x E^-08^ | 5.11 x E^-10^ | 4.16 x E^-08^ |
|  | Pb | 0.0002 | 2.16 x E^-10^ | 5.65 x E^-06^ | 5.15 x E^-10^ | 4.03 x E^-06^ | 1.01 x E^-09^ | 8.22 x E^-06^ | 2.40 x E^-09^ | 5.87 x E^-06^ |
| **Sharavati** | Mn | 0.0082 | 1.13 x E^-08^ | 2.94 x E^-04^ | 1.41 x E^-08^ | 1.47 x E^-05^ | 5.25 x E^-08^ | 4.28 x E^-04^ | 6.57 x E^-08^ | 2.14 x E^-05^ |
|  | Fe | 0.225 | 3.08 x E^-07^ | 8.04 x E^-03^ | 6.84 x E^-09^ | 2.68 x E^-05^ | 1.44 x E^-06^ | 1.17 x E^-02^ | 3.19 x E^-08^ | 3.90 x E^-05^ |
|  | Co | 0.0001 | 1.37 x E^-10^ | 3.57 x E^-06^ | 2.28 x E^-09^ | 1.19 x E^-05^ | 6.38 x E^-10^ | 5.20 x E^-06^ | 1.06 x E^-08^ | 1.73 x E^-05^ |
|  | Ni | 0.0002 | 3.01 x E^-10^ | 7.86 x E^-06^ | 5.57 x E^-11^ | 3.93 x E^-07^ | 1.40 x E^-09^ | 1.14 x E^-05^ | 2.60 x E^-10^ | 5.72 x E^-07^ |
|  | Cu | 0.0002 | 2.87 x E^-10^ | 7.50 x E^-06^ | 2.39 x E^-11^ | 1.88 x E^-07^ | 1.34 x E^-09^ | 1.09 x E^-05^ | 1.12 x E^-10^ | 2.73 x E^-07^ |
|  | Cd | - | - | - | - | - | - | - | - | - |
|  | Pb | 0.0002 | 3.01 x E^-10^ | 7.86 x E^-06^ | 7.17 x E^-10^ | 5.61 x E^-06^ | 1.40 x E^-09^ | 1.14 x E^-05^ | 3.34 x E^-09^ | 8.17 x E^-06^ |
| **Kali** | Mn | 0.004 | 5.47 x E^-09^ | 1.43 x E^-04^ | 6.84 x E^-09^ | 7.14 x E^-06^ | 2.55 x E^-08^ | 2.08 x E^-04^ | 3.19 x E^-08^ | 1.04 x E^-05^ |
|  | Fe | 0.0042 | 5.75 x E^-09^ | 1.50 x E^-04^ | 1.28 x E^-10^ | 5.00 x E^-07^ | 2.68 x E^-08^ | 2.18 x E^-04^ | 5.96 x E^-10^ | 7.28 x E^-07^ |
|  | Co | 0.0003 | 4.10 x E^-10^ | 1.07 x E^-05^ | 6.84 x E^-09^ | 3.57 x E^-05^ | 1.92 x E^-09^ | 1.56 x E^-05^ | 3.19 x E^-08^ | 5.20 x E^-05^ |
|  | Ni | 0.0005 | 6.84 x E^-10^ | 1.79 x E^-05^ | 1.27 x E^-10^ | 8.93 x E^-07^ | 3.19 x E^-09^ | 2.60 x E^-05^ | 5.91 x E^-10^ | 1.30 x E^-06^ |
|  | Cu | 0.002 | 2.74 x E^-09^ | 7.14 x E^-05^ | 2.28 x E^-10^ | 1.79 x E^-06^ | 1.28 x E^-08^ | 1.04 x E^-04^ | 1.06 x E^-09^ | 2.60 x E^-06^ |
|  | Cd | 0.0002 | 2.05 x E^-10^ | 5.36 x E^-06^ | 4.10 x E^-08^ | 1.07 x E^-05^ | 9.58 x E^-10^ | 7.80 x E^-06^ | 1.92 x E^-07^ | 1.56 x E^-05^ |
|  | Pb | 0.002 | 2.75 x E^-09^ | 7.18 x E^-05^ | 6.55 x E^-09^ | 5.13 x E^-05^ | 1.28 x E^-08^ | 1.05 x E^-04^ | 3.06 x E^-08^ | 7.47 x E^-05^ |
